# Preference of Chinese pre-service music teachers and schoolteachers for three culturally diverse musical pieces

**DOI:** 10.3389/fpsyg.2023.1297479

**Published:** 2024-01-26

**Authors:** Cancan Cui, Jin Liu, Yun Zhu

**Affiliations:** College of Music and Dance, Guangzhou University, Guangzhou, Guangdong, China

**Keywords:** pre-service music teachers, schoolteachers, culturally diverse music, personal preference, external preference

## Abstract

Using three musical pieces as musical stimuli from Romania, Brazil, and Saudi Arabia, this study extended Fung’s study by examining Chinese pre-service music teachers’ (*n* = 115) and schoolteachers’ (*n* = 131) personal preferences and external preferences for orchestral, folk, and popular music pieces. Two groups of participants were asked to select their preferred music from three pieces and to provide verbal descriptions of the reasons for their selections. The results showed (a) no significant differences in personal preference between the two groups but statistical significances in external preferences between the two groups; (b) the highest preference rating by both groups of teachers was *folk music* and the highest forced-choice preference was *popular music*; (c) statistically significant correlations between personal preference and external preference were evident in both groups across all three pieces; and (d) frequent descriptions by both groups of participants of the pieces in terms of musical characteristics were shared. The implications of this study are as follows. (1) Psychologists and music researchers gain insights about Chinese young adults’ listening preferences, which might offer implications for future research in the field of psychology; and (2) By enhancing multicultural awareness, music education teachers and researchers should combine different music styles to broaden students’ music horizons.

## Introduction

In the field of music, many psychologists and researchers have dedicated affective responses. Among the various aspects of affective response, scholars have devoted efforts to examining music preference, which is defined as “listeners’ liking for specific pieces of music as compared with others at particular points in time” (Hargreaves and Lamont, 2017). Many scholars and music teachers believe that expanding students’ music preference provides them with a broader perspective on the world and deepens their understanding of multiculturalism. In other words, an emphasis on music preference not only affects a person’s openness to other cultures, enhances their cross-cultural skills, and increases their respect of other cultures, but it would also function as a “springboard for further music learning” (Fung, 1995, p. 31; Lamont and Hargreaves, 2021; Dobrota et al., 2022; Kang et al., 2022, p.2).

According to the Tanglewood Symposia (I & II) (1967, 2007) and the National Standards (National Coalition for Core Arts Standards (NCCAS), 2014), music educators advocate for the importance of introducing students to a variety of music styles that are practiced worldwide (Yoo and Kang, 2020; Kang et al., 2022). In China, the updated National Arts Standard (2022), which was established by the Ministry of Education of the People’s Republic of China (2022), emphasized the need to listen to “foreign music,” with a purpose of raising students’ awareness of multiculturalism. With regards to music teaching and learning, students are encouraged to pay attention to Chinese music as well as respecting music from a variety of cultures. Therefore, studying music from diverse cultures is an indispensable step in the process of fostering students’ global perspectives.

Previous studies concentrated on investigating world music preferences among pre-service music teachers (Yoo et al., 2017; Kang et al., 2022). Most studies adopted multiple short excerpts rather than a few musical pieces in their entirety as musical stimuli (Confredo et al., 2020; Kang et al., 2022). Besides, researchers preferred to apply rating-scale approaches instead of a forced-choice approach (i.e., choosing one among the musical stimuli) (Herrera et al., 2018; Clark and Lonsdale, 2023; Cuadrado-García et al., 2023; Santos et al., 2023). To our knowledge, no study has compared Chinese pre-service music teachers’ and schoolteachers’ music preferences for pieces from outside China. Therefore, we extended Fung’s (2007) study to compare Chinese pre-service music teachers’ and schoolteachers’ personal preferences and external preferences for three culturally diverse musical pieces (Romanian orchestral piece, Brazilian folk piece, and Saudi Arabian popular piece). The three musical pieces were unfamiliar and remote from the participants’ culture. To clarify the scope, “culturally diverse music” for Chinese audiences was defined as music outside China.

## Literature review

Culturally diverse music can be viewed as “music from more than one culture and/or that beyond individual’s own culture” (Bennett, 2022). A substantial number of studies have examined culturally diverse music preferences along several dimensions (Fung, 1994, 1996, 2007; Morrison and Yeh, 1999; Yoo et al., 2017). Previous studies have asserted that participants prefer musical pieces with which they are familiar or that belong to their culture (Fung, 1996; Morrison and Yeh, 1999; Yoo et al., 2017). Researchers explored the relationships between musicians’ and non-musicians’ preferences for short instrumental excerpts from Africa, Asia, and Latin America with regards to the characteristics of the music. The findings indicated that participants preferred culturally familiar music pieces (Fung, 1996; Yoo et al., 2017). Morrison and Yeh (1999) further supported these results by exploring the aspects of Western music and Chinese music that influence participants’ preferences. They found that familiarity was one of the main factors that affected participants from mainland China and Hong Kong. Moreover, Fung (1994) found a moderate relationship between the world music preferences and the multicultural attitudes of non-music major undergraduate students.

When studying music preferences, only a few researchers have distinguished between personal preference and external preference (Fung, 2007; Fung et al., 2023). In line with Pratte (1992), personal preference refers to “a preference for the assignment of one set of goods (such as music) or opportunities over other sets of goods or opportunities for oneself” (p.39), while external preference can be defined as “a preference for the assignment of one set of goods or opportunities over other sets of goods and opportunities for others as well as for oneself” (p. 39). Pratte (1992) concluded that personal preference was related to, and possibly a component of, external preference and that personal preference was reflective of subjectively perceived values. Fung (2007) investigated the effects and interrelationships of five variables (*familiarity, perceived values, external preference for musicians and for K-12 students, and personal preference*) using three entire Chinese musical pieces (orchestral, traditional, and popular) among pre-service music teachers in the United States. Results indicated that pre-service music teachers preferred the orchestral piece, followed by the traditional piece and the popular piece. In addition, personal preference was consistently related to the perceived value of the music, external preference for musicians, and external preference for K-12 students [i.e., kindergarten (K) through twelfth grade (12)]. Recent study presented by Fung et al. (2023) explored the relationships between external preference and personal preference; again, a correlation was found between personal preference and external preference.

Moreover, individuals with distinctive backgrounds exhibited discrepant music preferences. Morrison and Yeh (1999) compared the music preferences of music and non-music major students from China, Hong Kong and the U.S. and found that participants from all three countries preferred musical pieces from their own culture. Musicians and non-musicians were also compared in Fung’s (1996) research, which showed that musicians exhibited higher preference ratings than non-musicians. Musicians preferred music pieces with relatively more complex textures (with multiple musical lines that are different), while non-musicians preferred moderately complex texture (Fung, 1996). Therefore, individuals with different cultural and musical backgrounds may exhibit distinct music preferences.

Limited number of research applied both rating-scale and forced-choice approach concurrently in measuring culturally diverse music preference within the recent decades. Forced-choice preference refers to “the rater chooses the items that most characteristics of the person rated” (Zavala, 1965, p.117). In the present study, listeners must pick one piece among the three pieces as the most preferred piece, and another one as the least preferred piece. Previous study indicated by applying both rating-scale and forced-choice approach to measure fifth graders’ music preferences, significant differences were found through forced-choice approach, yet no differences were exhibited through rating scale (Demorest and Schultz, 2004). However, Fung’s (2007) demonstrated an opposite result which indicated that both rating-scale and forced-choice approach demonstrated the same preference rank order.

Recent studies rarely investigated the descriptions of the music and reasons for preference. By examining adolescents’ musical preferences, and conducting follow-up semi-structured interviews with them, Kong (2023) found that participants adopted affective reactions such as the familiarity with the language or emotional feeling to describe their preferred musical piece. As for college students, they adopted analytical responses in describing their favorable musical piece among western classical music, jazz, and Chinese classical music (Morrison, 1998; Morrison and Yeh, 1999). Fung’s (2004) finding indicated that when describing their preferences of three traditional East Asian musical pieces, familiarity with the music was relatively unimportant; pre-service music teachers mostly described their preferences with analytical reasons, followed by affective and judgment reasons. Thus, there is a need to compare the verbal descriptions and reasons for preferences in the same study between pre-service music teachers and schoolteachers.

## Purpose and research question

Pre-service music teachers were the primary participants in previous music preference studies (Fung, 2007; Yoo et al., 2017). Some studies have included both musicians and non-musicians to examine their different music preferences (Hedden, 1974; Huber and Holbrook, 1980; Madsen and Geringer, 1981; Radocy, 1982; Sims, 1987). Other studies have compared the culturally diverse music preferences of musician and non-musician participants from China, Hong Kong and the U.S. with a focus on Asian pieces and found that cultural familiarity was one of the factors that affected participants’ choices (Morrison and Yeh, 1999). However, there is a lack of evidence regarding a distinction between pre-service music teachers and schoolteachers. This issue must be further explored and discussed. To our knowledge, no study has compared Chinese pre-service music teachers’ and schoolteachers’ music preferences for pieces from outside Asia. We extended Fung’s (2007) study of pre-service music teachers’ music preferences through three Chinese stimuli in different music genres to compare Chinese pre-service music teachers’ and schoolteachers’ personal preferences and external preferences for orchestral, folk, and popular music pieces from Romania, Brazil, and Saudi Arabia. The research questions were as follows: (1) What are the differences in five variables (*familiarity, perceived subjective value of music, external preference for musicians, external preference for K-12, and personal preference*) between the pre-service music teachers’ group and schoolteachers’ group across the Romanian orchestral piece, the Brazilian folk piece, and the Saudi Arabian popular piece? (2) To what extent does preference rating relate to forced-choice preference? (3) To what extent are pre-service music teachers’ and schoolteachers’ personal preferences related to familiarity, perceived subjective value of the music, external preference for musicians and external preference for K-12? (4) What are pre-service music teachers’ and schoolteachers’ verbal descriptions of the pieces? (5) What are the perceived reasons for pre-service music teachers’ and schoolteachers preferring a piece the most and the least? (6) What is the relationship between verbal descriptions and reasons for their preferences in each teacher group?

## Materials and methods

### Participants

Following approval from the university’s institutional review board, this study recruited 246 volunteer sophomore and junior level pre-service music teachers (*n* = 115) and schoolteachers (*n* = 131) (76.02% females, 23.98% males). The participants were enrolled in the same undergraduate elective course entitled “music teaching and activity guidance in elementary and secondary school” in fall 2022 and spring 2023 at a university in southern China. In each of these semesters, two classes were taught: one for music major students (students who select music as their primary degree concentration) and the other for non-music major students with a background in the field of education. The non-music majors were from 13 departments, including art education, physics education, chemistry education, math education, applied psychology, literature education, physical education, educational technology, history education, biological education, political science education, early childhood education, and English education. The course was for 16 weeks each semester.

### Musical stimuli

Three entire musical pieces were used as the musical stimuli which were selected from three culturally diverse music communities; these included an orchestral piece from Romania, a folk piece from Brazil and a popular music piece from Saudi Arabia. All three pieces were recommended by three different natives and representative of their respective cultures (see Table 1). Two music educators with expertise in culturally diverse music were invited to confirm the distinction and characteristics of each piece, including varied instrumentations, styles, and tempi, and their opinions were further confirmed by other members of the research team. The orchestral piece *(the skylark)* had a clear melody with varied orchestration at a fast and steady tempo. It featured the full Western orchestra, a solo violin, and a solo piccolo. In the middle of the piece, a modulation emerged. The folk choro piece (*Brasileirinho*) featured a guitar solo and Brazilian percussion ensemble. It began from A major with an entrance to B minor and back to A major, which displayed an A-B-A structure. The popular piece *(In Bold)* featured a male singer accompanied by an electric ensemble. Both the folk and popular music pieces were consistent with moderate tempos throughout. The three selected pieces ranged from 2′23″ to 3′19″ in duration. In addition, to verify the participants’ personal preferences and external preferences, we put considerable effort into ensuring that most of the participants were unfamiliar with the three pieces because evidence from previous research has shown that participants prefer pieces with which they are familiar (Fung, 2007; Yoo et al., 2017) or that represent their cultural identity (Morrison and Yeh, 1999). Lastly, rather than adopting short excerpts as found in most preference studies (Fung, 1996; Morrison and Yeh, 1999; Geringer and Guerra, 2002; Gürgen, 2015; Yoo et al., 2017), we applied the entire piece to examine participants’ preference, which was one of the features in the current study.

**TABLE 1:** Description of three pieces.

Style	Title of the piece	Performer	Composer/Arranger	Duration	Resource
Orchestral	Ciocarlia (The Skylark)	Barbu Lautaru Folk Music Orchestra	Anghelus Dinicu	2:23	Audio from Apple Music
Folk	Brasileirinho	Waldir Azevedo and Seu Conjunto	Waldir Azevedo	2:31	Audio from Apple Music
Popular (male)	Bel Bont El3areedh (In Bold)	Hussain Al Jassmi	Ayman Bahjat Qamar	3:19	Audio from Apple Music

### Measurement instrument

The participants took an online survey via Wjx.cn (similar to Qualtrics) that consisted of three parts: the consent form, demographic information, and Fung’s (2007) world music preference scale. Demographic information, including gender, major and minority status, was collected at the beginning of the instrument. Fung’s (2007) instrument was comprised of thirteen 7-point Likert scale items that included five variables (*familiarity, perceived subjective value of the music, external preference for musicians, external preference for K-12 students, and personal preference*). The first three items pertained to *familiarity* with music, followed by another three items of *perceived subjective value of music*. *External preferences* encompassed 6 items, with 4 items emphasized *external preference for musicians*, and 2 items focused on *external preference for K-12 students*. The last item emphasized *personal preference*. After the rating scale, there was an open-ended question that addressed the subjects’ verbal description of the piece. The measurement instrument demonstrated solid reliability, with Cronbach’s alphas of 0.86, 0.88, and 0.90 for the orchestral, traditional, and popular pieces, respectively (Fung, 2007). Since the participants listened to three different pieces, they filled out the same survey instrument three times, once for each music piece. Toward the end, the participants were asked to select their most preferred and least preferred musical piece across the three musical pieces and to provide a rational for their preferences to aid in the interpretation of their selection. In terms of Chinese participants, the first author, who is fluent with both English and Chinese, initially translated the instrument from English to Chinese. This was followed by a back translation that was completed by another bilingual music education doctoral candidate. The research team finally generalized and confirmed the sentence flow of the final version of the instrument, which enhanced the content validity of the survey instrument.

### Procedures

Convenience samples were drawn from four different classes, which were contacted by the instructor directly. The participants independently chose whether to take part. The students who agreed to participate remained in the classroom after the end of the class. After scanning the QR code displayed on the class monitor, the participants were initially asked to complete the electronic informed consent form on the first page in the survey as required by the university. The research team told the students that they were going to listen to three full pieces of music and respond to the same survey instrument questions three times. No title, composer, performer, cultural origin, or genre information was mentioned. All three musical pieces were resourced from and played through Apple Music, which guaranteed the sound quality of the music. After listening to each piece, the participants responded to the instrument over a 2-min interval. After listening to all three pieces, the participants responded to the final questions by choosing their most and least preferred piece across all three pieces and left a written response of the reasons for their preference choice. Finally, the research team performed a verbal investigation through a show of hands to investigate whether any participants had previously listened to any of the three pieces. The whole process took approximately 15 min.

## Results

The interitem correlations of each variable between groups were computed via SPSS 27. All coefficients were significant at the 0.01 level, with median coefficients ranging from 0.25 to 0.68 in the pre-service music teachers’ group and lower coefficients within each variable ranging from 0.04 to 0.67 in the schoolteachers’ group. We further calculated the internal consistency of each variable separately within each of the two groups. In the pre-service music teachers’ group, high internal consistency was exhibited, with Cronbach’s alpha values of 0.77, 0.80, and 0.78 for the orchestral, folk, and popular pieces, respectively. However, medium to high coefficient alphas of 0.57, 0.69, and 0.74 were found for the orchestral, folk, and popular pieces, respectively, in the schoolteachers’ group.

*RQ1: What are the differences in five variables between the pre-service music teachers’ group and schoolteachers’ group across the Romanian orchestral piece, the Brazilian folk piece, and the Saudi Arabian popular piece?*

Prior to examining group differences across the five dependent variables (*familiarity, perceived subjective value of music, external preference for musicians, external preference for K-12, and personal preference*), we determined that the data set violated the assumption of sphericity (Box *M* = 0.023, *p* < 0.05). An overall multivariate analysis of variance (MANOVA) was not appropriate for analysis. Therefore, each of the dependent variables was analyzed through a separate mixed-design ANOVA with one between-subjects variable (the two groups of pre-service teachers) and one within-subjects variable (the three musical pieces) (see Table 2). We used an alpha level of 0.01 (i.e., 0.05/5) based on Bonferroni correction for Type I error.

**TABLE 2:** Summary of analysis of variance procedures with familiarity, perceived subjective value of music, external preference for musicians, external preference for K-12 students, and personal preference.

Source	*SS*	*df*	*MS*	*F*	*p*
**Familiarity**					
Group	663.85	1	663.85	19.93	0.000
Musical pieces	93.46	2	49.78	7.09	0.001
Group × musical pieces	67.36	2	35.87	5.11	0.006
Error	3218.55	488	7.01		
**Perceived subjective value of music**					
Group	496.42	1	496.42	23.43	0.000
Musical pieces	136.21	2	71.57	14.70	0.000
Group × musical pieces	28.88	2	15.18	3.12	0.050
Error	2261.69	488	4.87		
**External preference for musicians**					
Group	808.68	1	808.68	20.48	0.000
Musical pieces	199.51	2	112.81	15.50	0.000
Group × musical pieces	37.52	2	20.99	2.91	0.062
Error	3141.65	488	7.28		
**External preferences for K-12 students**					
Group	73.74	1	73.74	7.20	0.008
Musical pieces	27.49	2	14.19	5.64	0.004
Group × musical pieces	15.01	2	7.74	3.08	0.049
Error	1188.85	488	2.51		
**Personal preference**					
Group	2.53	1	2.53	0.91	0.341
Musical pieces	25.15	2	12.89	10.75	0.000
Group × musical pieces	1.79	2	0.92	0.76	0.463
Error	570.77	488	1.17		

*N* = 246.

The within-subjects effect of the three musical pieces [*F*(2,488) = 7.09, *p* < 0.001] and the between-subjects effect of the two teacher groups [*F*(1,244) = 19.93, *p* < 0.0001] on *familiarity* were statistically significant (Greenhouse-Geisser). The interaction effect of groups by pieces on *familiarity* was also significant [*F*(2,488) = 5.11, *p* < 0.001]. *Post hoc* comparisons exhibited significant differences between (a) the orchestral piece and (b) the folk piece (*p* < 0.002) and the popular piece (*p* < 0.004) but not between the folk piece and the popular piece (*p* > 0.69). In addition, the results indicated that the popular piece (*M* = 9.31, *SD* = 0.26) displayed the highest mean score across the three musical pieces (*Mfolk* = 9.21, *SD* = 0.25; *Morchestral* = 8.51, *SD* = 0.24).

For the variable *perceived subjective value of music*, the main effect of the three musical pieces [*F*(2,488) = 14.70, *p* < 0.0001] and the main effect of the two groups of teachers [*F*(1,244) = 23.43, *p* < 0.0001] were statistically significant (Greenhouse-Geisser). However, no significant interaction effect was found between the groups and the three musical pieces [*F*(2,488) = 3.12, *p* > 0.01]. *Post hoc* comparisons of the three musical pieces displayed significant differences between (a) the popular piece and (b) the folk piece (*p* < 0.000) and the orchestral piece (*p* < 0.000) but not between the folk piece and the orchestral piece (*p* > 0.01). In addition, the results indicated that the orchestral music piece (*M* = 16.48, *SD* = 0.20) displayed a higher mean score than the folk (*M* = 16.21, *SD* = 0.20) and popular (*M* = 15.46, *SD* = 0.21) pieces for the *perceived subjective value of music* across the three musical pieces.

The main effects of the three musical pieces on *external preference for musicians* and *external preference for K-12* were statistically significant, with Greenhouse-Geisser [*F*(2,488) = 15.50, *p* < 0.0001] and [*F*(2,488) = 5.64, *p* < 0.01], respectively. Statistical significance was also found for the effects of grouping on the *external preference for musicians* [*F*(1,244) = 20.49, *p* < 0.000] and *external preference for K-12* [*F*(1,244) = 7.20, *p* < 0.008]. However, no significant interaction was found for the effect of grouping and the three musical pieces on either dependent variable. *Post hoc* comparisons of the *external preference for musicians* showed statistical significance between (a) the popular piece and (b) the folk piece (*p* < 0.000) and the orchestral piece (*p* < 0.000) but not between the orchestral piece and the folk piece (*p* > 0.01). Additionally, the mean score of the orchestral piece ranked the highest (*M* = 21.03, *SD* = 0.25) among the three musical pieces (*Mfolk* = 20.56, *SD* = 0.27; *Mpopular* = 19.77, *SD* = 0.28). *Post hoc* comparisons of *external preference for K-12* showed statistical significance only between the folk piece and the popular music piece (*p* < 0.002). In addition, the folk piece exhibited a higher (*M* = 9.71, *SD* = 0.15) mean score than the orchestral piece (*M* = 9.61, *SD* = 0.13) and the popular piece (*M* = 9.25, *SD* = 0.15).

Finally, the effects of the two groups of teachers and of the three pieces on *personal preference* were computed. The main effect of the three musical pieces on *personal preference* was statistically significant with Greenhouse-Geisser [*F*(2,488) = 10.75, *p* < 0.0001]. However, no significant difference (*p* > 0.05) was found between the two groups of pre-service teachers, and there was no significant interaction effect between the groups and the three musical pieces. *Post hoc* comparison of the three musical pieces showed significant differences between (a) the orchestral piece and (b) the folk piece (*p* < 0.000) and the popular piece (*p* < 0.007) but not between the folk piece and the popular piece (*p* > 0.09). In addition, among the three musical pieces (*Morchestral* = 4.35, *SD* = 0.75; *Mpopular* = 4.64, *SD* = 0.09), the mean score of the folk piece was the highest (*M* = 4.80, *SD* = 0.09).

*RQ2: To what extent does preference rating relate to forced-choice preference?*

**FIGURE 1:**
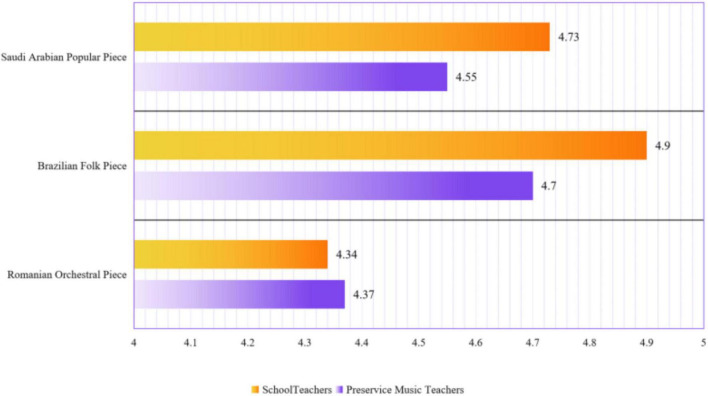
Mean scores of preference rating across three pieces in two groups.

Personal preferences across the three music pieces between the two groups were incongruent with forced-choice preferences, which yielded means of 4.37, 4.70, and 4.55 (orchestral, folk, and popular) in the pre-service music teachers’ group and means of 4.34, 4.90, and 4.73 (orchestral, folk, and popular) in the schoolteacher group (see Figure 1).

**FIGURE 2:**
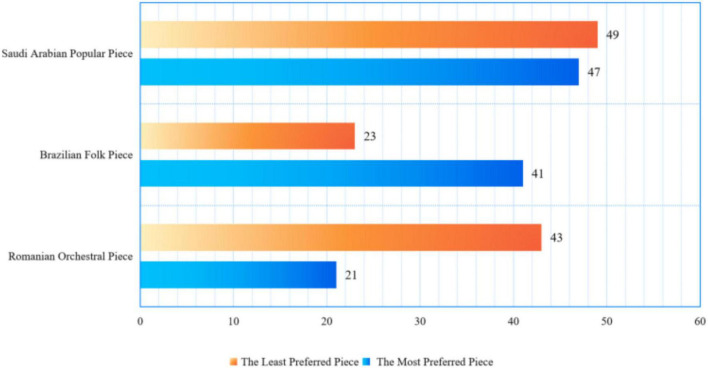
Results of the forced-choice preference in pre-service music teachers.

However, the results of forced-choice preferences showed that both groups of teachers preferred popular pieces the most (pre-service music teachers = 47, 40.8%; schoolteachers = 55, 41.9%), followed by Brazilian folk pieces (pre-service music teachers = 41, 35.6%; schoolteachers = 48, 36.6%) and then orchestral pieces (pre-service music teachers = 27, 23.4%; schoolteachers = 28, 21.3%) (see Figure 2).

**FIGURE 3:**
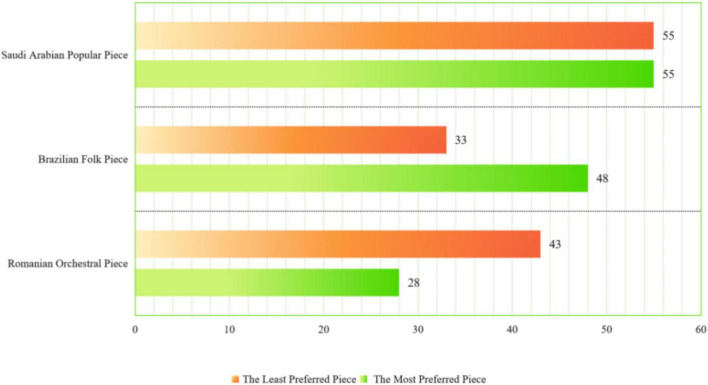
Results of the forced-choice preference in schoolteachers.

Interestingly, the least preferred piece was the popular piece (pre-service music teachers = 49, 42.6%; schoolteachers = 55, 41.9%), followed by the orchestral piece (pre-service music teachers = 43, 37.3%; schoolteachers = 43, 32.8%) and the folk piece (pre-service music teacher = 23, 20%; schoolteachers = 33, 25.1%) (see Figure 3). The results from the chi-square test displayed significant differences between the most preferred piece and the least preferred piece (chi-square = 128.95, *df* = 4, *p* < 0.0001). The results indicated that there was a weak correlation between the preference rating and forced-choice preference.

*RQ3: To what extent are pre-service music teachers’ and schoolteachers’ personal preferences related to familiarity, perceived subjective value of the music, external preference for musicians and external preference for K-12?*

Pearson correlation coefficients were determined to examine the relationship of the five variables (*familiarity, perceived subjective value of music, external preference for musicians, external preference for K-12 students, and personal preference*) in each musical piece and in each group. Tables 3–5 show that in the pre-service music teacher group, the five variables were consistently correlated with each other across all three pieces (*r* range: 0.25–0.72). Specifically, the strongest correlations were found between personal preference and perceived value in popular pieces (*r* = 0.72, *p* < 0.01), followed by folk pieces (*r* = 0.68, *p* < 0.01). The lowest correlations were exhibited between familiarity and perceived value across all three pieces (*r* range: 0.25 to 0.38). Low to modest interactions were found between personal preference and familiarity across all three pieces, ranging from *r* = 0.39 to *r* = 0.51. The relationships between personal preference and external preference for musicians were significant but modest across all three pieces, with *r* ranging from 0.49 to 0.57, *p* < 0.01. Personal preference was modest in relation to external preferences for K12, ranging from *r* = 0.56 to *r* = 0.64, *p* < 0.01 among all three pieces.

**TABLE 3:** Pearson correlation coefficients of Romanian Orchestral Piece for pre-service music teachers.

Romanian Orchestral Piece				
	**Familiarity**	**Perceived value**	**Ext. Pref. Mus.**	**Ext. Pref. K-12**
Perceived value	0.25**			
Ext. pref. mus.	0.41**	0.56**		
Ext. pref. k-12	0.40**	0.51**	0.65**	
Personal preference	0.51**	0.50**	0.57**	0.63**

**p < 0.01 (*n* = 115).

**TABLE 4:** Pearson correlation coefficients of Brazilian Folk Piece for pre-service music teachers.

Brazilian Folk Piece				
	**Familiarity**	**Perceived Value**	**Ext. Pref. Mus.**	**Ext. Pref. K-12**
Perceived value	0.32**			
Ext. pref. mus.	0.42**	0.65**		
Ext. pref. k-12	0.49**	0.56**	0.65**	
Personal preference	0.44**	0.68**	0.54**	0.64**

**p < 0.01 (*n* = 115).

**TABLE 5:** Pearson correlation coefficients of Saudi Arabian Popular Piece for pre-service music teachers.

Saudi Arabian Popular Piece				
	**Familiarity**	**Perceived value**	**Ext. Pref. Mus.**	**Ext. Pref. K-12**
Perceived value	0.38**			
Ext. pref. mus.	0.43**	0.61**		
Ext. pref. k-12	0.43**	0.62**	0.60**	
Personal preference	0.39**	0.72**	0.49**	0.56**

**p < 0.01 (*n* = 115).

In the schoolteachers’ group, statistical interactions were found across all three musical pieces. The strongest correlation was exhibited between personal preference and external preferences for K-12 (*r* = 0.70, *p* < 0.01), followed by personal preference and perceived value in the popular music piece (*r* = 0.67, *p* < 0.01). Relationships between personal preference and familiarity displayed the lowest correlation, ranging from 0.25 to 0.40. The lower values were also positioned between personal preferences and external preferences for musicians across all three pieces, ranging from 0.24 to 0.46. Additionally, lower and non-significant correlations were observed between familiarity and perceived value, external preference for musicians, and external preference for K-12 (*r* ranging from 0.04 to 0.16, *p* > 0.05) for the orchestral piece. For the folk piece, familiarity was not significantly related to perceived value and external preference for musicians (*r* ranging from 0.10 to 0.12, *p* > 0.05) (see Tables 6–8).

**TABLE 6:** Pearson correlation coefficients of Romanian Orchestral Piece for schoolteachers.

Romanian Orchestral Piece				
	**Familiarity**	**Perceived value**	**Ext. Pref. Mus.**	**Ext. Pref. K-12**
Perceived value	0.16			
Ext. pref. mus.	0.06	0.41**		
Ext. pref. k-12	0.04	0.43**	0.40**	
Personal preference	0.25**	0.47**	0.24**	0.50**

**Correlation is significant at the 0.01 level (2-tailed), *n* = 131.

**TABLE 7:** Pearson correlation coefficients of Brazilian Folk Piece for schoolteachers.

Brazilian Folk Piece				
	**Familiarity**	**Perceived value**	**Ext. Pref. Mus.**	**Ext. Pref. K-12**
Perceived value	0.13			
Ext. pref. mus.	0.10	0.52**		
Ext. pref. k-12	0.26**	0.56**	0.57**	
Personal preference	0.33**	0.58**	0.46**	0.70**

**Correlation is significant at the 0.01 level (2-tailed), *n* = 131.

**TABLE 8:** Pearson correlation coefficients of Saudi Arabian Popular Piece for schoolteachers.

Saudi Arabian Popular Piece				
	**Familiarity**	**Perceived value**	**Ext. Pref. Mus.**	**Ext. Pref. K-12**
Perceived value	0.30**			
Ext. pref. mus.	0.19*	0.61**		
Ext. pref. k-12	0.23**	0.64**	0.62**	
Personal preference	0.40**	0.67**	0.30**	0.52**

**Correlation is significant at the 0.01 level (2-tailed), *n* = 131.

*Correlation is significant at the 0.05 level (2-tailed), *n* = 131.

*RQ4: What are pre-service music teachers’ and schoolteachers’ verbal descriptions of the pieces?*

The research team applied an inductive approach to analyze the open-ended response of verbal descriptions of three musical pieces between the two groups (Patton, 2014). To identify the relationship between verbal descriptions and reasons for music preferences (i.e., the most and the least preferred pieces), the research team examined a total number of 1,899 comments (pre-service music teachers: 780 comments, schoolteachers: 1,119 comments) and identified emerging comment themes. Each pre-service music teacher and schoolteacher made from 1 to 75 open comments for each piece. Based on the participants’ comments, seven themes emerged in both groups of teachers: (1) musical characteristics (i.e., rhythm, melody, timbre, beat), (2) emotional feeling (i.e., exciting, expressive, happy, noisy, harsh, hot, danceable), (3) imagination or experiences (natural, bird singing, jungle, wide animal, bar), (4) musical styles (i.e., jazz, blues, samba, Indian, South-Asian, South-American, African, Thailand), (5) metaphorical connections (i.e., sounds like Tom and Jerry or sounds like musical drama), (6) music teaching and techniques (i.e., instrumental, voice technique, good for students), and (7) general comments (i.e., sounds good, like, dislike, not bad, unfamiliarity). The research team members agreed upon the categorization of the comments.

In the pre-service music teachers’ group, most of the participants used music terminology (164 comments) to describe the three musical pieces. Frequencies of emotional feeling (124 comments) ranked second in the teachers’ descriptions of their feelings across all three pieces, followed by general comments (91 comments). The frequencies of musical styles ranked fourth across all three pieces within this group, with 60 comments used to describe the inferred countries of all three pieces. Some pre-service music teachers described the three pieces with reference to their imagination or their past experiences (56 comments). Professional musical techniques and teaching ranked sixth, with an average of 21 comments. Finally, with 7 comments, the participants adopted films or movies as metaphors to describe the pieces that they heard (see Table 9).

**TABLE 9:** Frequencies of verbal descriptions in pre-service music teachers.

	Frequencies of verbal descriptions
Music characteristics	164
Emotional feeling	124
General comments	91
Musical styles	60
Imagination or experiences	56
Music teaching and techniques	21
Metaphorical connections	7

Verbal descriptions in the schoolteachers’ group were coded into similar themes but with different focuses according to their diverse backgrounds. Similar to the pre-service music group, most schoolteachers frequently used music terminology (231 comments) to describe the three musical pieces, with 94 comments for the popular piece and 66 comments for the orchestral piece. Ranked second was the descriptions of their individual emotional feelings (220 comments), with 85 comments, 83 comments, and 52 comments were found for folk piece, orchestral piece, and popular piece, respectively. Imagination or past experiences (126 comments) ranked third, followed by musical styles (60 comments), and general comments (31 comments). Professional music teaching and metaphorical connections ranked last, with 27 comments each (see Table 10).

**TABLE 10:** Frequencies of verbal descriptions in schoolteachers.

	Frequencies of verbal descriptions
Music characteristics	231
Emotional feeling	220
Imagination or past experiences	126
Musical styles	60
General comments	31
Music teaching and technique	27
Metaphorical connections	27

*RQ5: What are the perceived reasons for pre-service music teachers and schoolteachers preferring a piece the most and the least?*

In terms of choosing the most preferred piece, music characteristics (49 comments) ranked the highest in the pre-service music group, followed by general comments (39 comments) and emotional feeling (24 comments). However, when choosing the least preferred musical piece amongst the three pieces, general comments (60 comments) ranked the highest, followed by music characteristics (59 comments). It’s worth noting that metaphorical connections showed minimal occurrences as reasons explaining why a piece was chosen as the most preferred (1 comment) or the least preferred piece (1 comment) in the pre-service music teachers’ group.

In the schoolteachers’ group, emotional feeling (65 comments) ranked the highest reasons for choosing the most preferred piece, followed by music characteristics (61 comments) and general comments (44 comments). However, when choosing the least preferred piece amongst the three pieces, music characteristics (83 comments) ranked the highest, and general comments (76 comment) ranked second. Notably, professional music teaching (4 comments) and metaphorical connections (1 comment) had the fewest occurrences as the reasons for choosing the most and least preferred piece, respectively.

*RQ6: What is the relationship between verbal descriptions and reasons for their preferences in each teacher group?*

Results indicated that pre-service music teachers frequently used musical elements, emotional feelings, and general comments to describe the pieces they heard, whereas schoolteachers mostly described the three pieces using musical elements, emotional feelings, and their imagination and personal experiences. In addition, the most notable reasons for preferences were the music characteristics in both groups. Other than music characteristics, both groups of participants incorporated their feeling with music in describing the reasons for preferring a specific type of music. In summary, the relationship between verbal descriptions and reasons for preferences of the music was somewhat parallel in pre-service music teachers’ group, yet the relationship was not parallel in schoolteachers’ group.

## Discussion and conclusion

We sought to replicate and extend Fung’s (2007) study and investigated personal preferences and external preferences across three entire musical pieces (Romanian orchestral, Brazilian folk, and Saudi Arabian popular) between pre-service music teachers and schoolteachers in China. In particular, we first explored the differences in five variables between the two groups across three musical pieces, followed by examining the relationship between preference rating and forced-choice preference in the two groups. Furthermore, we attempted to determine relationships among pre-service music teachers’ and schoolteachers’ personal preferences related to familiarity, subjective perceived value of the music, and external preferences for musicians and K-12 students. Open-ended responses of verbal descriptions and perceived reasons were further transcribed, coded, and analyzed.

The primary results indicated that there was no difference between the pre-service teachers’ and the schoolteachers’ personal preferences. This result contrasted with those of Fung (1996), as his study revealed a significant difference between the music group and the non-music group. Several potential reasons might explain these results. First, as we presented above, most schoolteachers had taken the “music teaching and activity guidance in elementary and secondary school” course for 16 weeks, and the contents of the course were abundant and dynamic, encompassing rhythm, melody, improvisation, movement, music appreciation, choral conducting, minority music from China, and culturally diverse music outside China. These contents increased their exposures to different music genres and music activities, which may have influenced their personal preferences (Peretz et al., 1998; McKoy, 2002; Gürgen, 2015; Chmiel and Schubert, 2019). Additionally, music activities such as karaoke and live house are abundant everywhere, which encourages more individuals to show their passion and capacity for music and pursue their favorite style of music (Law and Ho, 2015). These reasons may have contributed to the lack of significant differences between the pre-service music teachers and the schoolteachers.

However, there were group differences in two variables (external preference for musicians and external preference for K-12 students), and these differences may be attributed to the personal backgrounds of the two groups of teachers. Although both groups of teachers have educational backgrounds, the different primary degree concentration might explain schoolteachers’ unfamiliarity with the conditions and purpose of what a musician should perform on stage or what a K-12 music teacher should teach in school. Even though the schoolteachers had completed a music course and being trained as music specialists, the course had a limited duration. Most schoolteachers lacked practical experience in music teaching since they were still sophomore and junior-year college students who paid more attention to their own majors. Given these reasons, we inferred that even though they showed personal interest in music, they still lacked a deep understanding of music subjects (Holden and Button, 2006).

The results drawn from the relationship between preference rating and forced-choice preference were inconsistent. Initially, in the preference rating, both groups of teachers rated the *folk music piece* the highest, whereas in the forced-choice preference, the majority of the participants in both groups selected the *popular music piece* as their most preferred. The different measurement methods used may have contributed to this inconsistent finding. In many existing studies, a rating scale has been applied to test individuals’ music preferences (Fung et al., 2000; Gürgen, 2015; Yoo et al., 2017; Kang et al., 2022). However, the forced-choice approach has been seldomly used in music listening studies when compared to the rating scale (Demorest and Schultz, 2004; Fung, 2007), especially within the recent decades. Therefore, based on Fung’s (2007) study and the current findings, the forced-choice preference should be brought to other researchers’ attention for future use.

Another result which emerged from the forced-choice preferences was that both groups of teachers selected *popular music* as both their “most preferred” and their “least preferred” type of music. To further clarify, the participants were anonymous, therefore, the total number of participants who chose folk and orchestral pieces as their most preferred music might be the same participants who disliked the popular piece in both groups. In other words, the popular piece was polarized, indicating that the participants either liked it or disliked it, and there was not much middle ground. It is common for participants to select the popular music piece as their most preferred piece (Clark and Lonsdale, 2023; Santos et al., 2023). This is because the openness policy, an open window issued by the Chinese government, has inspired an increasing number of Chinese students to pursue interests in their preferred popular music from inside or outside China (Fung et al., 2000; Ho, 2017). Due to the openness policy, a large portion of foreign popular cultures worldwide have entered China’s culture, which has impacted the Chinese music market. In this way, the exposure of students to popular music has increased (Landsberger, 2009). As Law and Ho (2015) observed, the majority of Chinese students enjoy international popular music. They are fond of music not only from China but also from outside the country (Clark and Lonsdale, 2023). Another possible influence would be the “idol” culture. A large group of students’ popular music preferences is influenced by their “idol” (Ho, 2017). Fondness for Chinese, American, Japanese, and Korean artists have become a typical trend of youth culture on the mainland. This trend has encouraged more young Chinese students to access the music of their idols’ latest musical recordings (Fung, 2008; Cockrill and Lui, 2013). For some students, their popular music preferences are based on their “idol’s” songs, music preferences, and singing techniques (Ho, 2014). Regardless of what their idols sing, the Chinese students’ music preferences follow (Ho, 2014). As the most preferred music, popular music was also the least preferred music in the current study. A potential reason for this could be individual personality, which is an underlying factor that is associated with music preference but is not included in this study (Langmeyer et al., 2012; Vella and Mills, 2017; Herrera et al., 2018). Langmeyer et al. (2012) revealed that personality might be a predictor of music preference, as participants who disliked pop music could be introverted. Therefore, we speculated that the participants who put popular music as their least preferred piece might be Introverted.

Evidence of correlations among the five variables between the two groups across three musical pieces indicated that familiarity was moderately correlated with personal preference. This result was partly aligned with the results from Fung’s (2007) study, which indicated a moderately significant relationship between familiarity and preference for a popular piece. However, in the current study, moderate and significant correlations between familiarity and personal preference for the orchestral piece and the folk piece, respectively, were also observed. We speculate that the potential reasons might be related to the current advancement of social media and music applications (Hu et al., 2021). Fung’s study was published in 2007, when technology and internet resources were limited. However, technology is now more advanced, and electronic resources and mobile apps are partially free for users. It is very convenient for Chinese students to connect with different music from within and outside China through the internet, internet-connected devices, or mobile apps (Law and Ho, 2015). The innovation of the internet and social media platforms, such as TikTok, Spotify, Apple Music have tremendously influenced Chinese students’ music preferences and practices (Fung, 2009; Zhang and Mao, 2013; Law and Ho, 2015; Hu et al., 2021). Users can use their personal devices to directly download digital audio and video files that help students appreciate music more conveniently. Due to the enormous transformation of the internet in urban China in the last decade, young Chinese students can now effortlessly access a wide range of music genres online (Cockrill and Lui, 2013; Hu et al., 2021). In this way, the lives and lifestyles of many young Chinese people, as well as their preferences for music, have been affected by advanced technology and the internet (Fung, 2009; Zhang and Mao, 2013; Hu et al., 2021).

The results confirmed the relationships between personal preferences and external preferences in both groups of teachers, which was in line with the results from Fung’s (2007) study. Nevertheless, in the current study, the relationship between personal preference and external preference for K-12 students was consistently higher than the relationship between personal preference and external preference for musicians, not only across three pieces, but also in both groups. We inferred that first, both pre-service music teachers and schoolteachers believed that it is more important for K-12 to gain perspectives of culturally diverse music than professional musicians. Second, since all the participants in the current study had educational backgrounds and possibly K-12 working experiences, they paid more attention to elementary and secondary school teaching. These findings contrast with the results from Fung’s (2007) research indicating the relationship between personal preference and external preferences for musicians were higher than that with external preference for K-12 students.

Both pre-service music teachers and schoolteachers frequently described the music pieces through music characteristics (Boyle et al., 1981; Fung, 1994, 2007; Morrison and Yeh, 1999). As expected, all participants in the present study had music experiences of varying durations. The schoolteachers had taken music lessons for 16 weeks, which allowed them to describe music pieces through simple music terminology. However, schoolteachers who acquired advanced music backgrounds or music knowledge were able to illustrate their thoughts through accurate music terminology when describing music characteristics such as melody, mood, rhythm, and speed. Following music characteristics, most pre-service music teachers described their feelings through emotional references, whereas music teachers used some general comments to describe the pieces they heard. We speculated that this might be attributed to differences in their major area of study. Music teachers have numerous opportunities to be exposed to music listening activities or participate in music listening surveys; thus, they take it for granted that they can adopt simple and general comments to describe the pieces they have heard, while schoolteachers lack opportunities to take part in music listening surveys. Once they have the opportunity, they express their emotional feelings carefully. We also discovered that both groups of participants preferred to use metaphorical comments to illustrate the pieces (Morrison and Yeh, 1999). Between the two groups, schoolteachers made more metaphoric comments than pre-service music teachers, and we inferred that this might be because schoolteachers are more word-savvy than pre-service music teachers.

### Implications

Implications can be drawn for psychologists, scholars, educators, and students in the realm of music. Through investigating young adults’ personal preference and external preference by listening to culturally diverse music, psychologists and scholars could potentially predict the lifestyles (North and Hargreaves, 2007a), leisure activities (North and Hargreaves, 2007b), personality (Herrera et al., 2018), motivations (Chin et al., 2018), and emotions (Nieminen et al., 2012) through the lens of music.

Another insight involves supporting researchers in the field of music, psychology, and music industry. Results from the present study reflected that *popular music* was the young adults’ the “most preferred” and the “least preferred” type of music. By understanding this “polarized result,” music psychologists and researchers could explore young adults’ psychological status and establish new measurement instruments or incorporate advanced technologies (i.e., ECG) in conducting music preference research. In addition, based on the “polarized result,” music businessperson could gain potential insights about music industry development by consulting music psychologists and music researchers.

Through exploring culturally diverse music preferences, the participants gained insights into music from various cultural contexts, which not only broadening students’ musical horizons, but also further influenced their awareness of multiculturalism, especially since most participants in the current study possess educational backgrounds. In the future, when they are dealing with their own students, their perspective and wisdom of cultural diversity might have an impact on selecting music to incorporate into their teaching which would deepen their influences on their students.

### Limitations

The limitation of the current study was the limited number of musical pieces. Only three musical pieces were adopted as the stimulus due to the participants’ attention span, memory and patience. As a feature of the study, we used the entire piece of music which is rare in preference studies (Fung, 2004, 2007; Philibotte et al., 2023), rather than short excerpts which is commonly used in preference studies (Fung, 1996; Morrison and Yeh, 1999; Geringer and Guerra, 2002; Gürgen, 2015; Yoo et al., 2017). Due to the application of the entire pieces, there is a need for participants to spend a much longer time to receive the musical stimulus. It would have been challenging for participants if more pieces were used; that is due to the fact that participants would need more time to listen to the entire piece, and they may forget the first piece after they hear the last one.

### Future research

Three aspects can be addressed in future research explorations. One potential direction is to add more amount of music in different genres and from diverse cultures to further investigate Chinese participants’ music preferences. Furthermore, as a feature of the current study and considering the memory and psychological status, we played three entire pieces from three diverse cultures. In our future research, numbers of short excerpts might apply to strengthen the diversity and variability of cultural diversity, music styles, and music genres, and a comparison study between short excerpts and entire songs might be needed.

Another possible research direction might be to investigate the emphasis of external preference in music preference. To date, only a few studies have concentrated on the analysis of external preferences toward music preferences (Pratte, 1992; Fung, 2007; Fung et al., 2023), which confirmed the interrelationships between personal preferences and external preferences among pre-service and in-service music teachers in the United States. Participants from other cultural contexts need to be explored further. Thus, future research exploring the relationship between external preference, other possible variables, and the effect of external preferences on several dependent variables (e.g., music teaching and learning, personality, emotion) can be further reviewed.

Third, future research should consider increasing the sample size. Our current study had a limited number of diverse participants. In our future research, we will aspire to recruit participants with diverse backgrounds, not only educational backgrounds but also non-educational and nonmusical backgrounds. Their demographic backgrounds such as age, SES, music background and time length of learning music might be further taken into account as a control variable for prediction or other analyses.

## Author’s note

I appreciated all the suggestions and feedback from Dr. C. Victor Fung.

## Data availability statement

The raw data supporting the conclusions of this article will be made available by the authors, without undue reservation.

## Ethics statement

This study was approved by the Institutional Review Board from Guangzhou University. The studies were conducted in accordance with the local legislation and institutional requirements. The participants provided their signed electronic informed consent to participate in this study.

## Author contributions

CC: Conceptualization, Data curation, Formal analysis, Investigation, Methodology, Project administration, Software, Validation, Writing—original draft, Writing—review and editing. JL: Project administration, Supervision, Validation, Writing—original draft, Writing—review and editing. YZ: Data curation, Formal analysis, Validation, Writing—review and editing.
